# Management of Acute Coronary Syndromes in Patients in Rural Australia: The MORACS Randomized Clinical Trial

**DOI:** 10.1001/jamacardio.2022.1188

**Published:** 2022-05-25

**Authors:** Fiona Dee, Lindsay Savage, James W. Leitch, Nicholas Collins, Conrad Loten, Peter Fletcher, John French, Natasha Weaver, Olivia Watson, Helen Orvad, Kerry J. Inder, Dawn McIvor, Trent Williams, Allan J. Davies, John Attia, John Wiggers, Aaron L. Sverdlov, Andrew J. Boyle

**Affiliations:** 1 John Hunter Hospital, Department of Cardiovascular Medicine, Hunter New England Local Health District, Newcastle, New South Wales, Australia; 2 School of Nursing and Midwifery, College of Health, Medicine and Wellbeing, University of Newcastle, Callaghan, New South Wales, Australia; 3 School of Medicine and Public Health, College of Health, Medicine and Wellbeing University of Newcastle, Callaghan, New South Wales, Australia; 4 Hunter Medical Research Institute, New Lambton, New South Wales, Australia; 5 John Hunter Hospital, Department of Emergency Medicine, Hunter New England Local Health District Newcastle, New South Wales, Australia; 6 Liverpool Hospital, South Western Sydney Local Health District, Liverpool, New South Wales, Australia; 7 South Western Sydney Clinical School, University of New South Wales, Sydney, New South Wales, Australia; 8 Tamworth Rural Referral Hospital, Hunter New England Local Health District Tamworth, New South Wales, Australia; 9 Population Health, Hunter New England Health Local Health District, Newcastle, New South Wales, Australia

## Abstract

**Importance:**

Treatment of ST-segment elevation myocardial infarction (STEMI) in rural settings involves thrombolysis followed by transfer to a percutaneous coronary intervention–capable hospital. The first step is accurate diagnosis via electrocardiography (ECG), but one-third of all STEMI incidents go unrecognized and hence untreated.

**Objective:**

To reduce missed diagnoses of STEMI.

**Design, Setting, and Participants:**

This cluster randomized clinical trial included 29 hospital emergency departments (EDs) in rural Australia with no emergency medicine specialists, which were randomized to usual care vs automatically triggered diagnostic support from the tertiary referral hospital (management of rural acute coronary syndromes [MORACS] intervention). Patients presenting with symptoms compatible with acute coronary syndromes (ACS) were eligible for inclusion. The study was conducted from December 2018 to April 2020. Data were analyzed in August 2021.

**Intervention:**

Triage of a patient with symptoms compatible with ACS triggered an automated notification to the tertiary hospital coronary care unit. The ECG and point-of-care troponin results were reviewed remotely and a phone call was made to the treating physician in the rural hospital to assist with diagnosis and initiation of treatment.

**Main Outcomes and Measures:**

The proportion of patients with missed STEMI diagnoses.

**Results:**

A total of 6249 patients were included in the study (mean [SD] age, 63.6 [12.2] years; 48% female). Of 7474 ED presentations with suspected ACS, STEMI accounted for 77 (2.0%) in usual care hospitals and 46 (1.3%) in MORACS hospitals. Missed diagnosis of STEMI occurred in 27 of 77 presentations (35%) in usual care hospitals and 0 of 46 (0%) in MORACS hospitals (*P* < .001). Of eligible patients, 48 of 75 (64%) in the usual care group and 36 of 36 (100%) in the MORACS group received primary reperfusion (*P* < .001). In the usual care group, 12-month mortality was 10.3% (n = 8) vs 6.5% (n = 3) in the MORACS group (relative risk, 0.64; 95% CI, 0.18-2.23). Patients with missed STEMI diagnoses had a mortality of 25.9% (n = 7) compared with 2.0% (n = 1) for those with accurately diagnosed STEMI (relative risk, 13.2; 95% CI, 1.71-102.00; *P* = .001). Overall, there were 6 patients who did not have STEMI as a final diagnosis; 5 had takotsubo cardiomyopathy and 1 had pericarditis. There was no difference between groups in the rate of alternative final diagnosis.

**Conclusion and Relevance:**

The findings indicate that MORACS diagnostic support service reduced the proportion of missed STEMI and improved the rates of primary reperfusion therapy. Accurate diagnosis of STEMI was associated with lower mortality.

**Trial Registration:**

anzctr.org.au Identifier: ACTRN12619000533190

## Key Points

**Question:**

Does a decision support service improve diagnosis of ST-segment elevation myocardial infarction (STEMI) in rural hospitals without emergency medicine specialists?

**Findings:**

In this randomized clinical trial, 29 rural hospitals in Australia were assigned to usual care vs system-initiated centralized decision support (management of rural acute coronary syndromes [MORACS] intervention). Missed STEMI diagnosis was associated with higher mortality and was significantly more frequent in the usual care group, with missed diagnoses of STEMI virtually eliminated in the MORACS group and administration of primary reperfusion therapy increased.

**Meaning:**

The findings show that MORACS diagnostic support reduced the proportion of missed STEMI diagnoses and increased rates of primary reperfusion therapy.

## Teaser

This randomized clinical trial seeks to improve outcomes of ST-segment elevation myocardial infarction in rural areas in Australia by reducing missed diagnoses and increasing administration of primary reperfusion therapy.

## Introduction

In rural and regional settings, reperfusion therapy for ST-segment elevation myocardial infarction (STEMI) involves thrombolysis followed by transfer to a percutaneous coronary intervention (PCI)–capable hospital.^1,2^ Rapid and correct diagnosis is the first step in the chain of survival for patients with STEMI. Significant gains have been made in using electrocardiograph (ECG) transmission platforms for prehospital diagnosis by ambulance paramedics; however, one-third of patients presenting directly to Australian hospital emergency departments (EDs) with STEMI do not receive primary reperfusion treatment. The causes of this remain incompletely understood.^3,4^

In the SNAPSHOT-ACS^3^ study, failure to treat STEMI occurred in 152 of 421 patients (36.1%). Failure to treat in the acute phase resulted in an increase in mortality (18 [11.7%] vs 13 [4.9%]; *P* = .01).^4^ In rural New South Wales, Australia, a missed diagnosis of STEMI, defined as failure to identify and initiate treatment within 4 hours, is predominantly due to failure to correctly interpret ECG and serum markers, and this is seen mainly in rural hospitals without specialists.^5^ The Management of Rural Acute Coronary Syndromes (MORACS) trial was developed to test a system-triggered real-time diagnostic support service for rural hospitals with the aim of preventing missed diagnoses of STEMI.

## Methods

### Study Design

Ethics approval was obtained through the Hunter New England Health Service Human Research Ethics Committee. This trial was performed in accordance with the principles of the Declaration of Helsinki and followed the Consolidated Standards of Reporting Trials (CONSORT) reporting guideline.^6^ Informed consent was obtained from hospital executives but not for individual patients, as they were randomized by hospital to usual care or MORACS.

**Figure 1.**
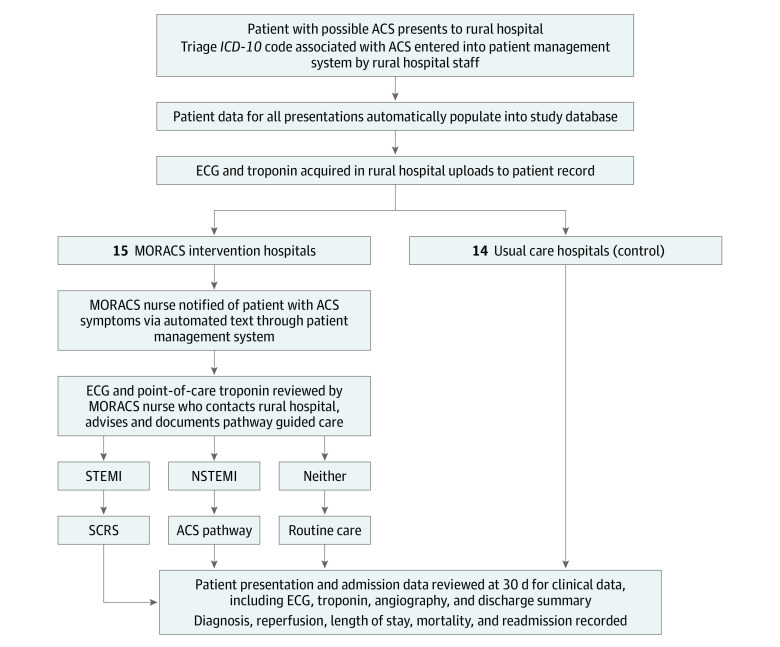
Study Design for the Management of Rural Acute Coronary Syndromes (MORACS) Randomized Clinical Trial ACS indicates acute coronary syndrome; ECG, electrocardiography; *ICD-10*, *International Classification of Diseases, Tenth Revision*; NSTEMI, non–ST-segment elevation myocardial infarction; SCRS, state cardiac reperfusion strategy; STEMI, ST-segment elevation myocardial infarction.

The MORACS trial was a prospective multisite cluster randomized clinical trial that aimed to evaluate the effectiveness of a centralized acute coronary syndrome (ACS) diagnostic support system to improve health outcomes for patients in rural settings. A centralized system was adopted for the study as a recognized economically efficient and effective way of delivering specialized clinical care and reducing clinical variation across large geographical areas.^7^ An overview of the approach is presented in Figure 1.

MORACS was conducted within the Hunter New England local health district (LHD) in New South Wales, Australia. Rural hospital EDs with no emergency medicine specialists (ie, EDs staffed with general practitioners or career medical officers) were included. All EDs across the LHD (including intervention and usual care sites) are equipped with ECG carts and defibrillators that use the Glasgow algorithm to provide acceptable automated diagnostic support in the recognition of myocardial ischemia (including STEMI) in conjunction with opt-in specialist review (cardiologist, emergency specialist, or cardiology advanced trainee) as part of the New South Wales cardiac reperfusion strategy. ECG results meeting STEMI criteria are transmitted to the established opt-in support service strategy (24-hour/7-day services based at John Hunter Hospital and Tamworth Rural Referral Hospital), which has been in place in the LHD for more than 12 years.

### Inclusion and Exclusion Criteria

**Figure 2.**
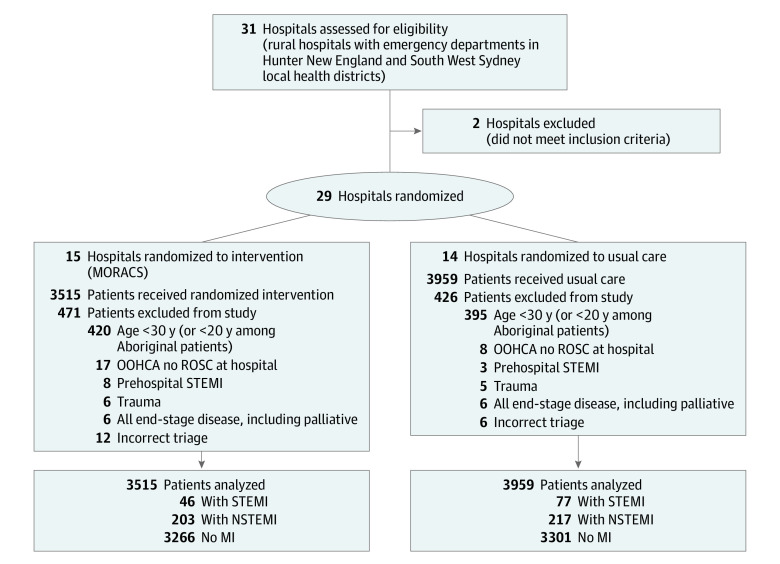
Flow Diagram in the Management of Rural Acute Coronary Syndromes (MORACS) Randomized Clinical Trial MI indicates myocardial infarction; NSTEMI, non–ST-segment elevation myocardial infarction; OOHCA, out-of-hospital cardiac arrest; ROSC, return of spontaneous circulation; STEMI, ST-segment elevation myocardial infarction.

From December 2018 to April 2020, all patients triaged with *International Statistical Classification of Diseases and Related Health Problems*, *Tenth Revision *(*ICD-10*) codes relevant to possible ACS, including I20.9, I.20.0, R07.4, I46.9, R10.1, R10.4, and R06.0^8^ in the ED of the 29 rural hospitals within the LHD were considered eligible for the study. For a complete list of included *ICD-10* codes, see eTable 4 in the Supplement. Data from ED presentations with these codes were automatically populated to the MORACS database. All consecutive patients presenting to the hospital with suspected ACS (eg, chest pain for investigation or other heart-related symptoms, such as shortness of breath, or who are triaged as possible ACS) were included. Presentations to intervention hospitals instigated via an automatic system-generated text message notification to the MORACS nurse. Recruitment and follow-up data are summarized in Figure 2.

Non-Indigenous patients younger than 30 years and Indigenous patients younger than 20 years were excluded, given the low likelihood of ACS in these populations,^6^ as well as patients with prehospital ECG scans performed by ambulance paramedics showing STEMI, as these patients were treated via the existing state cardiac reperfusion pathway, including diversion where possible to the nearest PCI-capable facility. Indigenous status is collected via self-report on admission in all Australian hospitals. Data on indigenous status were included in this study because coronary events have been found to occur at an earlier age in Indigenous populations in Australia.^9,10^ Also excluded were patients who were treated palliatively who would not have been offered reperfusion, those with cardiac arrest with no return of spontaneous circulation, or those with traumatic injuries with a clear noncardiac cause. Clinicians within rural hospitals randomized to the MORACS intervention were educated regarding the intervention to enable implementation and engagement; clinicians in the usual care hospitals were not. Hospital discharge, ECG scans, troponin, and coronary angiography reports were used to confirm the final diagnosis.

The initial research proposal included South West Sydney LHD to demonstrate scalability; however, this LHD was subsequently excluded owing to the appointment of ED specialists. The rationale for including hospitals staffed by general practitioners and career medical officers was that higher proportions of missed myocardial infarction were previously reported at these hospitals.^5^ MORACS was resourced with 1 specialist nurse per day providing decision-making support from a central hub within the LHD.

Using the existing medical record system, an automated, system-triggered, real-time electronic notification system was developed to alert the MORACS specialist nurse when a patient was triaged with symptoms suggestive of ACS at the rural hospital. All ECG scans across the LHD EDs automatically download to the patient’s electronic medical record for review on acquisition. This is enabled through a gateway that integrates to the LHD cardiac picture archive and communication system. Results of point-of-care troponin are also available in the patient’s electronic medical record.

This facilitated real-time review of the ECG (to diagnose or rule out STEMI) and troponin level (for assessment of ACS) remotely by MORACS nurses and enabled review of all suspected ACS cases in the usual care hospitals for adjudication of STEMI. Importantly, this did not require the rural hospital clinicians to initiate contact and did not replace established pathways. Contact was then initiated by the MORACS nurse to the local treating clinicians at the rural hospital. The clinical situation and results were discussed, and the patient was triaged into 1 of 3 existing clinical pathways—STEMI, non-ST elevation ACS, or noncardiac management—per the treating clinician.

Twenty-nine rural hospitals without emergency medicine specialists staffing the ED were randomized to either usual care (n = 14) or the MORACS decision-support intervention (n = 15) (eTable 1 in the Supplement). These rural hospitals support an estimated population of 218 436 covering a region of approximately 100 000 square kilometers (38 600 square miles) with bed numbers that range from 0 (multipurpose services with first aid post only) to 100.^11,12^ Randomization was stratified based on hospital size and medical staffing (general practitioners vs career medical officers).

Indigenous people represent 7.1% of the overall population within this LHD.^13^ Coronary angiography is not available any of the rural hospitals included in the study, and therefore patient transfer by road ambulance is required (which occurs in most cases) unless weather or patient condition requires transfer by air.

There are 2 PCI-capable public hospitals within the LHD: John Hunter Hospital, a tertiary referral hospital in Newcastle providing a 24-hour, 7-day per week primary PCI service, and Tamworth Rural Referral Hospital, which offers a 3-day-per-week service during business hours. Transport distances to a primary PCI-capable hospital can exceed 600 km (370 miles).

### MORACS Intervention

The MORACS team comprised 3 specialist clinical nurses with expertise in ECG interpretation and treatment of patients with suspected ACS. The MORACS intervention operated between 9:00 am and 7:30 pm 7 days per week with 1 nurse rostered per day.

The MORACS service nurses were notified of patients with suspected ACS presenting at intervention hospitals via a text message generated through the patient information management system (iPM [DXC Technology]) (Figure 1). Intervention hospital clinicians could contact the MORACS team directly by phone to review patients in the ED with suspected ACS. This process was developed to ensure that clinicians could seek further advice where required for patients already identified through MORACS, and identify new patients initially triaged with an alternative *ICD-10* code. An opt-in support system has been in place for more than 12 years where rural hospitals can contact the tertiary referral hospital for clinical advice. The MORACS service removed the requirement for the treating clinician to initiate this contact during operation hours.

When patients presented outside of MORACS hours, they were treated by rural clinicians as per the established pathway (including the opt-in support service) and reviewed by the MORACS nurse at the commencement of the shift (9:00 am). The relevant intervention hospital was contacted if the patient remained in the hospital or in the event a patient had been or was being discharged and required further assessment.

To quantify resource utilization, the time taken for the clinical review by the tertiary hospital clinician was recorded in a subset of patient encounters (n = 50). This included the time to review the ECG and troponin in the electronic medical record, the telephone consultation, and documentation into the research database.

### Outcomes

The primary outcome measure was the proportion of missed STEMI. STEMI was defined using ECG and clinical presentation criteria from the Fourth Universal Definition for Myocardial Infarction^14^ and in accordance with the Australian Guidelines for the Management of ACS.^15^ Missed STEMI was defined by failure to recognize STEMI on the ECG and initiate treatment for STEMI according to Australian guidelines. Recommended time to reperfusion with thrombolysis (unless contraindicated) from presentation to hospital is 30 minutes.^15,16^ If STEMI was not considered within a 4-hour period from presentation, the diagnosis was classified as missed.^5^ Missed STEMI events not already identified in the hospital admission documentation by a cardiologist were identified by ECG criteria of STEMI, which were then adjudicated by 2 cardiologists who were blinded to randomization.

Patients deemed eligible for immediate STEMI treatment (reperfusion) were those who presented to the hospital within 12 hours of symptom onset as per national and international guidelines.^2,15^ Secondary outcomes included in-hospital, 30-day, and 12-month mortality; time to reperfusion (for STEMI); 30-day readmission; and length of hospital stay. Data were analyzed in August 2021.

### Statistical Analysis

The sample size calculation was based on approximately 6300 presentations per year with suspected ACS in the rural hospitals included in the study. An estimated 2% to 5% of these presentations would be STEMI based on Australian data,^15^ with 36% of STEMI diagnoses being missed.^3^ Calculations indicated that 280 participants (140 per arm at 80% power and α = .05) were required to demonstrate an absolute reduction of 16% in the rate of missed STEMI diagnoses. A design effect of 1.24 (assuming cluster sizes of 13 and an intraclass correlation coefficient of 0.015) was used, which projected 15 months of recruitment based on the available number of clusters for the 2-year project.

Categorical data for all patients recruited to the study and those diagnosed with STEMI (including baseline characteristics of individual patients and primary and secondary outcomes) were summarized using frequency counts and percentages. Continuous variables were summarized using means and SDs for reporting age at presentation and medians and IQRs for time-related numerical variables (ie, time to reperfusion and length of stay). Continuous and categorical variables were compared using χ^2^ tests. A Kaplan-Meier survival analysis was performed for patients with STEMI comparing survival and correct diagnosis vs missed diagnosis in the MORACS intervention vs usual care. Comparison of survival distribution for MORACS, usual care correct diagnosis, and usual care missed diagnosis was performed using a log-rank test and Bonferroni correction adjusting for 3 groups. Relative risk of mortality and correct diagnosis vs incorrect diagnosis was reported at 12 months for the MORACS intervention compared with usual care. Statistical analyses were performed in SAS version 9.4 (SAS Institute) and SPSS version 24 (IBM). A 2-sided *P *value less than .05 was considered statistically significant.

## Results

A total of 7474 ED presentations with suspected ACS were recorded among 6249 patients (mean [SD] age, 63.6 [12.2] years; 3000 individuals [48%] were female). In the usual care group, 521 individuals (16%) were Indigenous and in the MORACS group, 414 individuals (14%) were Indigenous. See eTable 2 in the Supplement for entire patient cohort characteristics. STEMI was the final diagnosis in 77 patients (2.0%) in the usual care hospitals and 46 (1.3%) in the MORACS hospitals. Median (IQR) follow-up was 617 (495-743) days. Baseline characteristics of all patients are presented in Table 1; patients with STEMI are presented in Table 2. Extended patient characteristics are presented in eTable 3 in the Supplement. The median (IQR) time taken for each patient review by the tertiary hospital MORACS clinician was 12 (10-15) minutes, including the medical record review, phone call, and documentation.

**Table 1.** Baseline Characteristics at Presentation^a^

Characteristic	No. (%)	
	Usual care (n = 3342)	MORACS intervention (n = 2907)
Female	1639 (49)	1361 (47)
Male	1703 (51)	1546 (53)
Age at presentation, mean (SD), y	60.8 (16.3)	58.9 (16.1)
Indigenous	521 (16)	414 (14)
ASGS regionality (2016)		
Major cities	409 (12)	123 (4.2)
Inner regional	1451 (43)	1862 (64)
Outer regional	1457 (44)	908 (31)
Remote	16 (0.5)	3 (0.1)
Very remote	3 (0.1)	1 (0.0)
IRSAD 2016 quartile		
Quartile 1	1012 (30)	117 (4)
Quartile 2	2211 (66)	2725 (94)
Quartile 3	92 (2.8)	40 (1.4)
Quartile 4	21 (0.6)	15 (0.5)
Prior myocardial infarction (12 mo)	467 (14)	374 (13)
Prior coronary stent	317 (9.5)	277 (9.5)
Prior CABG	141 (4.2)	122 (4.2)
Diabetes	511 (15)	373 (13)

Abbreviations: ASGS, Australian Statistical Geography Standard; CABG, coronary artery bypass graft; IRSAD; Index of Relative Socio-economic Advantage and Disadvantage.

^a^ There were 7474 presentations in 6249 individual patients.

**Table 2.** Clinical Outcomes

Patients with STEMI (n = 124^a^)	No. (%)		
	Usual care (n = 77)^b^	MORACS intervention (n = 46)	*P* value
Female	17 (22)	10 (22)	NA
Male	60 (78)	36 (78)	NA
Age at presentation, mean (SD), y	65.1 (12.8)	61.2 (10.7)	NA
Indigenous	14 (18)	4 (9)	NA
Prior myocardial infarction (12 mo)	2 (3)	2 (4)	NA
Prior coronary stent	6 (8)	5 (11)	NA
Prior CABG	2 (3)	2 (4)	NA
Diabetes	18 (23)	9 (20)	NA
ED presentation in prior 21 d	7 (9)	1 (2)	NA
ED presentation during MORACS hours	57 (74)	32 (70)	NA
Mode of arrival			
Ambulance	20 (26)	10 (22)	NA
Private car	57 (73)	36 (78)	NA
Other	1 (1)	0	NA
Triage category			
ATS 1 resuscitation	2 (3)	3 (7)	NA
ATS 2 emergency	69 (88)	40 (87)	NA
ATS 3 urgent	4 (5)	3 (7)	NA
ATS 4 semiurgent	3 (4)	0	NA
ATS 5 nonurgent	0	0	NA
Infarction location			
Anterior	33 (43)	20 (44)	NA
Nonanterior	44 (57)	26 (57)	NA
Primary outcome			
Missed STEMI	27 (35)	0	<.001
Secondary outcomes			
In-hospital mortality	6 (7.7)	1 (2.2)	.20
30-d mortality	6 (7.7)	3 (6.5)	.81
12-mo mortality	8 (10.3)	3 (6.5)	.48
Hospital length of stay, median (IQR), d	4 (3-6)	3 (2-4)	.049
30-d Readmission (all cause)	10 (13)	11 (24)	.11
Primary reperfusion strategy			<.001
Thrombolysis and transfer	37 (47)	35 (76)	
Reperfusion ineligible	2 (3)	8 (17)	
Transfer for primary PCI	11 (14)	1 (2)	
Spontaneous reperfusion	1 (1)	2 (4)	
No reperfusion therapy (eligible cases)	27 (35)	0 (0)	
Time to reperfusion, median (IQR), min	74 (39-123)	55 (34-76)	.07
Admission and transfer	69	46	NA
Final diagnosis			
MI with obstructive coronary artery disease	64 (83)	44 (96)	NA
Takotsubo cardiomyopathy	4 (5)	1 (2)	NA
Spontaneous coronary artery dissection	0	1 (2)	NA
Pericarditis	1 (1)	0	NA
No definitive diagnosis/coronary angiography	8 (10)	0	NA

Abbreviations: ATS, Australian Triage Scale; CABG, coronary artery bypass grafting; ED, emergency department; MI, myocardial infarction; PCI, percutaneous coronary intervention; STEMI, ST-segment elevation myocardial infarction.

^a^ χ^2^ Test used for variable hypothesis tests.

^b^ One patient in the usual care group presented twice with STEMI.

The primary end point of the study, the proportion of missed STEMI, occurred in 27 of 77 patients (35%) in usual care hospitals and 0 of 46 (0%) in MORACS hospitals (*P* < .001). Of those eligible for primary reperfusion, 48 of 75 patients (64%) in the usual care group and 36 of 36 (100%) in the MORACS group received reperfusion therapy (*P* < .001) (Table 2). There were no statistically significant differences in reperfusion time, length of stay, or 30-day readmission rates (Table 2). Overall, there were 6 patients who did not have STEMI as final diagnosis: 5 had takotsubo cardiomyopathy and 1 had pericarditis (Table 2). There was no difference between groups in the rate of alternative final diagnosis. See eTables 5 and 6 in the Supplement for other outcomes.

**Figure 3.**
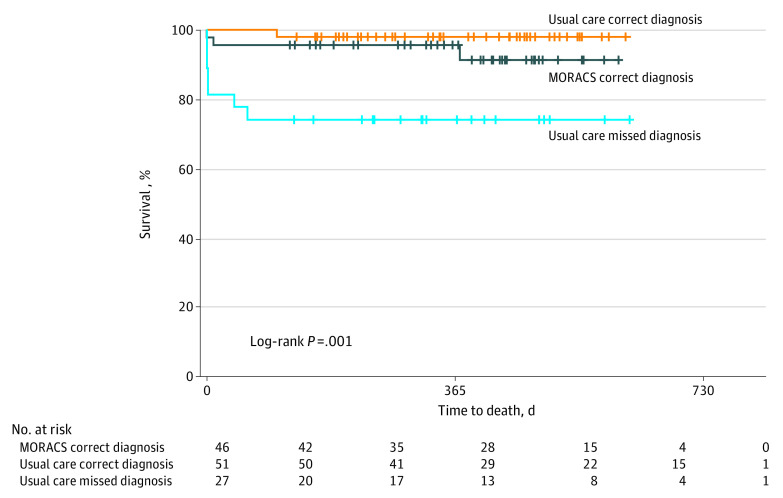
Mortality Associated With Missed Diagnosis of ST-Segment Elevation Myocardial Infarction (STEMI) in the Management of Rural Acute Coronary Syndromes (MORACS) Randomized Clinical Trial

Mortality at 12 months was 6.5% (n = 3 of 46) in the MORACS intervention group vs 10.3% (n = 8 of 77) in the usual care group (relative risk, 0.64; 95% CI, 0.18-2.23; *P* = .48). Within the usual care group, patients with a missed STEMI diagnosis had a mortality of 25.9% (n = 7 of 27) compared with 2.0% (n = 1 of 51) with a correct diagnosis (relative risk, 13.2; 95% CI, 1.71-102.00; *P* = .001), underscoring the importance of an accurate early diagnosis (Figure 3).

## Discussion

This multisite, cluster randomized clinical trial compared a centralized ACS diagnostic support service (MORACS) with usual care, with the primary aim of reducing the proportion of missed STEMI in non–specialist-managed rural hospital EDs. Findings demonstrate that a centralized diagnostic support service significantly reduced the proportion of missed STEMI. Accurate diagnosis of STEMI resulted in higher rates of initiating primary reperfusion. Further, a correct STEMI diagnosis was associated with lower mortality.

A rapid and accurate diagnosis is crucial in the treatment of patients with STEMI. International guidelines recommend that an ECG be performed within 10 minutes of first medical contact.^2,15,17^ However, rural physicians infrequently encounter STEMI and may not be experienced in ECG interpretation compared with physicians in tertiary referral cardiac units. This study has shown that a diagnostic support mechanism helped improve the accuracy of ECG interpretation. Despite all ECG machines in the district having automated algorithms to assess for STEMI with acceptable diagnostic accuracy in interpretation of STEMI, one-third of STEMI incidents remain undiagnosed.^18^

We found a large effect with our intervention, larger than projected, despite a lower rate of STEMI than anticipated (ie, 1.7% of suspected ACS presentations compared with 2% to 5% in prior studies^19^). Prehospital STEMI data during the study period indicate there were 116 additional patients diverted from MORACS rural and regional areas to either Tamworth or John Hunter Hospitals as per ambulance protocol. In addition to the 11 patients who were diagnosed with STEMI prehospital (Figure 2), this may account for this lower rate in hospital. Prehospital ECG and triage by ambulance paramedics have demonstrated excellent outcomes.^20,21^ By removing patients with STEMI with prehospital treatment, our trial was underpowered for mortality, although the point mortality estimates of 6.5% and 10.3% are in keeping with mortality rates following fibrinolytic therapy and when a significant proportion of patients do not receive pharmacologic reperfusion.^3,21^ However, in post hoc analyses within the usual care group, those with a correct diagnosis of STEMI did have lower mortality than those without, which is consistent with the evidence.^4^

While guidelines recommend time to reperfusion with thrombolysis from presentation to hospital of less than 30 minutes,^1,15^ other studies have found this time frame is often exceeded.^3^ Our mean time to reperfusion with thrombolysis from presentation to hospital was outside the recommended guidelines and should be the subject of future clinical improvement strategies.

The complete absence of missed STEMI diagnoses in the MORACS intervention group, including those presenting after hours, was unexpected. Possible explanations for this may include behavior change in the intervention hospitals owing to the MORACS service or the small number of patients observed (n = 14) in the cohort; nevertheless, this was statistically and clinically significant. Eleven patients in the usual care group and 1 in the MORACS group (who presented outside of MORACS hours) received primary PCI; however, it was unclear why this strategy was chosen in place of thrombolysis in the absence of documentation of contraindications to thrombolysis.

This model for supporting diagnosis and management of ACS may be easily adapted and scaled across a variety of health care environments. Further assessment of outcomes and economic evaluation for the remaining patients recruited will provide additional insights regarding the potential benefits of the MORACS intervention for all patients with suspected ACS (including those without STEMI).

### Limitations

There are several limitations to this study. First, the MORACS intervention was not provided 24 hours per day due to cost constraints within the trial. Future clinical rollout of this model should consider the need for after-hours coverage. Second, data on who made the STEMI diagnosis for patients in MORACS hospitals (ie, MORACS or rural hospital clinicians) were not collected, and therefore an assessment regarding whether recognition of STEMI by rural clinicians improved over time could not be completed. Third, postdischarge medication adherence and resource utilization associated with the intervention were not collected, but could be the subject of a data linkage program.

There are considerable strengths to this cluster randomized study—a large, inclusive study with clustering by hospital. The service can easily be applied to many other health care systems as our study was conducted across a large geographic area, serving diverse populations, and recorded the time required by the MORACS nurses to assist clinicians with decision-making for each patient. This would allow adaptation and upscaling of this protocol to other regions and services.

## Conclusions

The MORACS diagnostic support service significantly reduced the proportion of missed STEMI and improved the rates of primary reperfusion therapy. Accurate early diagnosis of STEMI was associated with lower mortality.

## Notes

Supplement 1. Trial protocol

Supplement 2. eTable 1. Hospitals included in the MORACS Trial eTable 2. All patients baseline characteristics eTable 3. STEMI patient demographics eTable 4. Triage codes used for recruitment in MORACS eTable 5. Primary and secondary outcomes eTable 6. Additional outcomes – reperfusion

Supplement 3. Data sharing statement
