# Supplementary material for: Management of Acute Coronary Syndromes in Patients in Rural Australia: The MORACS Randomized Clinical Trial
Source: JAMA Cardiol. 2022 May 25;7(7):690–8. doi: 10.1001/jamacardio.2022.1188 (PMC10881213; doi:10.1001/jamacardio.2022.1188)
Supplement: Supplement 1. — Trial protocol [file jamacardiol-e221188-s001.pdf]

Clinical Trial Protocol

Management Of Rural Acute Coronary Syndrome
(MORACS)

OVERVIEW	
Chief Investigator:	Professor Andrew Boyle
Host Organisation	John Hunter Hospital, Hunter New England Local Health District
Project title	Management Of Rural Acute Coronary Syndrome (MORACS)
Project summary	One third of patients presenting to Australian hospitals with ST-segment-Elevation Myocardial Infarction (STEMI) do not receive primary reperfusion treatment, which is the standard of care (1, 2). In rural and regional Australia, primary reperfusion treatment involves thrombolysis which, if delivered in timely fashion, provides outcomes similar to primary percutaneous coronary intervention (PCI) (3). Failure to provide reperfusion doubles mortality, increases length of stay and increases readmission rates (2). A high level of clinical variation, due to misdiagnosis or treatment of acute coronary syndromes (ACS), has been found in NSW. Our research in Hunter New England Health Local Health District (HNELHD) shows that missed acute myocardial infarction (MI) from failure to correctly interpret electrocardiogram (ECG) and serum markers, is common leads to increased mortality and morbidity, and is overrepresented in rural hospitals that are maintained by GPs (4).<br><br>This proposal will randomize small rural hospitals to either usual care or a mandated transmission of ECG and troponin from spoke (rural) hospitals to a central hub service. Protocol directed advice will be given to the randomized rural hospital clinicians, and will channel patients into the existing ACS management structures: The State Cardiac Reperfusion Strategy (SCRS) for STEMI patients, and the NSW Chest Pain Pathway for non-STEMI patients. The primary outcome will be the difference in identification of STEMI in patients who present to rural hospitals.<br><br>A cost-effectiveness evaluation will report the resources required to increase STEMI identification. Secondary outcomes include major adverse cardiac events (MACE) and outcomes in non-STEMI. HNELHD has a proven track record in innovation in rural cardiology. If successful and cost-effective, this project will define a new paradigm for assessment and management of ACS in rural settings,

	utilising a central clinical decision support service. It can rapidly be scaled up to be applied across NSW.
List all sites in which the project will be conducted	Rural hospitals in Hunter New England Local Health District:<br>Armidale, Barraba, Bingara, Cessnock, Denman, Dungog, Glenn Innes, Gloucester, Gunnedah, Guyra, Kurri Kurri, Merriwa, Moree, Murrurundi, Muswellbrook, Narrabri, Nelson Bay, Singleton, Scone, Tenterfield, Vegetable Creek, Walcha and Warialda;

Background and research question

Acute coronary syndromes (ACS), comprising both ST elevation myocardial infarction (STEMI) and non-ST elevation myocardial infarction (non-STEMI) are a significant cause of morbidity, mortality and cost to the community. In fact, ACS are the leading cause of mortality in Australia (5). In 2013-14 there were 104,100 hospitalisations for ACS (5). Direct hospitalisation costs are estimated at $1.8 billion, with total economic costs around $17.9 billion annually (6). People in rural and remote areas are 60% more likely to be hospitalised with ACS than people in major cities, and the mortality rate is also higher (5). Rural patients hospitalised for ACS often require hospital transfers, which add further burden on the ambulance and hospital systems and local community. Timely access to evidence-based and optimal ACS management, in both the acute and chronic phases of ACS, is imperative for optimal clinical care (7).

The failure to treat ACS, in particular STEMI, is a significant issue nationally. In the Snapshot-ACS study, failure to treat STEMI occurred in 36.8% of patients (1). Failure to treat in the acute phase resulted in a doubling of mortality (2). This preventable mortality, due to clinical variation, was not able to be more accurately quantified within the study design of Snapshot-ACS, but there are clearly medical, social and economic costs associated with suboptimal treatment of ACS.

Local causes for failure to treat STEMI in HNELHD have been collected after a systematic review of ACS avoidable deaths from the Clinical Excellence Commission in NSW. Local data has been systematically collected to better identify the problems inherent in treating ACS in the rural environment (4). The data shows a high incidence of misdiagnosis of STEMI in smaller, rural hospitals with a consequent increase in mortality. The missed acute myocardial infarction (MAMI) data indicates that failure to interpret the ECG and confused clinical judgment are the most frequent causes of failure to treat STEMI, occurring in 70% and 65% of missed STEMI cases respectively (4). We therefore propose to support diagnosis and clinical decision making in smaller rural hospitals to prevent missing the diagnosis of STEMI.

Aims: To improve identification and management of patients with ACS in rural hospitals across 3 LHDs by implementing a hub and spoke ACS management system.

Research Question: In patients admitted to a rural hospital with suspected ACS, does a centralised management system improve identification of STEMI and subsequent clinical outcomes in all ACS patients?

Hypotheses:

1. The MORACS program will improve identification of STEMI
2. The MORACS program will improve 30-day mortality, 12-month mortality, time to reperfusion, length of hospital stay and readmission rates in patients with suspected ACS presenting to rural hospitals

Our proposal addresses a variation in clinical care that is deadly, costly, potentially preventable. Hub-and-spoke models have been shown to be effective in other clinical situations, but our application is a novel use of this approach. Our telehealth solution to this clinical issue will maximise the clinical support to many rural hospitals.

The NSW State Cardiac Reperfusion Strategy (SCRS) aims to deliver increased access to medical advice and reperfusion therapies in a timely manner for patients diagnosed with STEMI. Our approach is complementary to the SCRS and will in no way overlap with this. We aim to assist clinicians earlier in the diagnostic work-up, to prevent the diagnosis being missed. If a diagnosis of STEMI is made, the patient will be treated via the usual SCRS process.

Research design and methods (maximum 4 pages)

Study design: A prospective, cluster randomised controlled trial (RCT) to evaluate the effectiveness of a hub and spoke ACS management system to improve health outcomes for rural residents.
Setting: Hunter New England Local Health District in regional/rural NSW Australia. The following hospitals will be included:
Armidale, Barraba, Bingara, Cessnock, Denman, Dungog, Glenn Innes, Gloucester, Gunnedah, Guyra, Kurri Kurri, Merriwa, Moree, Murrurundi, Muswellbrook, Narrabri, Nelson Bay, Singleton, Scone, Tenterfield, Vegetable Creek, Walcha and Warialda;

These hospitals were selected because General Practitioners (GPs) or Career Medical Officers (CMOs) manage cardiology admissions, i.e. there are no Emergency Medicine specialists at these hospitals. Together they assess over 6000 chest pain presentations per year. The rationale for including GP and CMO staffed hospitals was that these were the hospitals with higher numbers of missed myocardial infarctions in our prior study (4).

Participants: All patients presenting with chest pain for investigation to hospitals from rural and remote communities throughout the HNELHD. Notably, patients with prehospital ECGs performed by ambulance paramedics showing STEMI will NOT go through this process, they will go via existing SCRS pathways. All patients presenting to Emergency Departments, whether by self-transport or via ambulance without a prior STEMI diagnosis, will be included.

Eligibility: Inclusion criteria – Hospitals where GPs or Career Medical Officers (CMOs) manage ACS presentations will be included. All consecutive patients presenting to hospital with suspected ACS (eg. chest pain for investigation or other heart related symptoms such as shortness of breath or who are triaged as possible ACS) will be included.
Exclusion criteria – Hospitals staffed by Emergency Medicine specialists (FACEMs) will not be included. Our data suggest the rate of missed STEMI in these hospitals is low. There will be no patient-level exclusion criteria.

Randomisation: Hospitals will be randomised to receive the intervention (MORACS) or control (usual care). Randomisation will be undertaken independently by personnel from the HMRI statistical support unit. Randomisation will be stratified based on hospital size and medical staffing. Rural hospitals included in the study have bed numbers that range from 0 to 100. In most rural hospitals admissions are managed by on-call GPs where the GP attends as required. In a small number of rural hospitals admissions are managed by CMOs who are staffed 24 hours per day, therefore potentially able to offer more timely treatment. GP vs CMO staffing will be separately stratified to better identify different outcomes of care driven by differing medical management system in place.

Allocation to treatment:
Intervention arm - All patients presenting to an intervention hospital will have an ECG and serum troponin level result transmitted to a central reporting service based at John Hunter Hospital. This service is monitored by the Department of Cardiology at John Hunter Hospital. Staff with clinical expertise from the central reporting service will review the ECG and serum troponin level and determine the likelihood of an acute coronary syndrome. The central reporting service will contact the hospital with diagnostic support and advice on which evidence-based pathway to follow – the SCRS for STEMI or the NSW Chest Pain Pathway for non-STEMI. This utilises existing evidence-based pathways, rather than providing *ad hoc* advice on individual patient management. The service will be staffed by the clinical cardiac liaison officer of John Hunter Hospital on weekday morning shifts and by a dedicated afternoon shift and weekend clinical nurse consultant. Overnight will be staffed by the John Hunter CCU charge nurse. There is considerable experience at John Hunter Hospital with reading pre-hospital ECGs and discussing cases with the ambulance personnel who transmitted the ECG, as part of the SCRS. This project will extend this approach into rural hospitals and will also incorporate the results of troponin testing, thereby capitalising on existing expertise. This will facilitate expert ECG reading, even when the algorithm does not detect STEMI, as well as helping clinicians identify high risk ACS.

Control arm- Will continue with current practice and treatment processes which will consist of management of patients without supportive expert advice, except where this is requested as part of normal process.

Communication between referral hospital and central reading service: Using the iPIMS patient registration system, an automated notification to the reading clinician is generated when anyone is triaged with diagnoses compatible with acute coronary syndromes. All ECGs and point of care troponins are available on the centralised medical record (Clinical Applications Portal - CAP). The reading clinician will contact the rural hospital emergency department and will discuss the case with the treating clinician, whilst able to simultaneously view the results of troponin and ECG on CAP.

Follow-up: All patients will have outcomes data recorded at hospital discharge or hospital transfer in the medical record. Thirty-day and 12-month readmission rates (and reasons) will be identified through the Centre for Health Record Linkage (CHeReL). Deaths will be identified through the National Death Index. No individual patient follow-up will be performed.

Outcomes:
Primary Outcome: Proportion of correctly identified STEMI. ECGs will be adjudicated by a senior clinician at John Hunter Hospital in consultation with the treating clinician who has assessed the patient. All ECGs performed on all patients presenting with possible ACS will be analysed. In HNELHD, this will be performed via CAP online.

Secondary outcomes: In-hospital, 30-day and 12-month mortality, time to reperfusion (for STEMI), length of hospital stay, MACE (major adverse cardiac events: death, repeat MI or all-cause readmission) at 30-days and 12-months. Post discharge health care utilisation and medication adherence will likely be improved by better hospital care, so we will assess this by MBS and PBS linkage. Myocardial infarction will be defined by elevated troponin in the context of typical ischaemic symptoms. Mortality will be ascertained from the National Death Index (NDI). Readmissions will be captured by CHeReL. Reperfusion and revascularisation data will be ascertained from the Cardiac and Stroke Outcomes Unit in HNE.

Measures: Primary and secondary outcome data will be obtained from medical records and from CHeReL. We have discussed the project and received a formal quote from CHeReL. We will also link with MBS and PBS to assess post-discharge healthcare utilisation and medication prescription/adherence after hospitalisation. This will contribute valuable qualitative information as well as informing cost effectiveness analysis.

Statistical methods and analysis:
1) Power calculation: Based on local data, anticipated number of presentations to hospital with suspected ACS over a 12-month period are approximately 6,300. Of these an estimated 50% will require admission and investigation for ACS. Around 15% of the total patient group will have non-STEMI and a further 5% have STEMI, i.e. ~315 STEMI per year across all centres. An estimated 36% of the patients with STEMI will be missed and we wish to detect an absolute reduction in missed STEMI of 16%, i.e. 36% down to 20%, which will require 280 participants (140 per arm), at 80% power and p=0.05. We also include a design effect of 1.24 (assuming cluster sizes of 13 and ICC=0.015) for a total of 338 patients across 26 clusters total. Therefore, it will take approximately 15 months to recruit. In the two-year timeframe, we will be able to analyse the rate of STEMI detection (primary endpoint) and will have a *median* 12-month follow-up for the secondary endpoints.
2) Evaluation of Results: Analysis of the primary outcome (the proportion of correctly identified STEMI) will be via logistic regression with adjustment for stratifying variables (size and staffing of hospital), and analysis of the secondary outcomes will be via linear and logistic regression.
3) Economic evaluation: Assuming an improvement in STEMI identification, three economic evaluations will be conducted. 1) From a health provider perspective, a cost-effectiveness analysis to report the resources required per unit improvement in the identification of STEMI. Costs will include the cost of the intervention as well as related costs such as hospital stay,

treatments and readmissions. The measure of effect will be based on the primary outcome (identification of STEMI). 2) A cost-consequence analysis will report the cost of the intervention and its range of consequences such as the change in STEMI identification, reperfusion times, hospital admissions, readmissions and MIs. 3) A modelling exercise to estimate costs and potential downstream impacts from the intervention over a ten-year time frame, these will include avoided healthcare costs as a consequence of improved STEMI detection and treatment, as well as adherence to secondary prevention therapies. This will be informed by data linkage with MBS and PBS.

Considerations about Aboriginal and Torres Strait Islander Peoples: Aboriginal and Torres Stair Islander Peoples have disproportionately higher rates of ACS, and these occur at a younger age than in non-Indigenous people (8). In rural settings, Aboriginal and Torres Strait Islander Peoples make up a higher percentage of the population. Our project has been thoroughly discussed with and reviewed by the Aboriginal Health Unit of the HNE LHD. Improving diagnosis, management and outcomes for rural ACS patients will disproportionately advantage Aboriginal and Torres Strait Islander Peoples.

Implications for Ambulance: NSW ambulance service (NSWAS) have been consulted and are a partner for this project. Existing ambulance work flows will not be changed. With increased diagnosis of STEMI, there will be a higher rate requirement for urgent transport to a Primary PCI centre, under the SCRS. It is estimated this will be an additional 60 STEMI patients over the 15 months recruitment period. Ambulance will benefit from earlier notice of transfer in non-STEMI patients, which will improve workflow. Ambulance have requested data on outcomes to inform their practice, and we will provide data on ambulance transported patients back to NSWAS.

Governance structure: The project will be supervised by the MORAC steering committee. The steering committee will comprise project investigators, partner representatives, clinicians, operational managers, clinical stream and clinical governance representatives and will meet bi-monthly.
There will be two distinct working groups operating under the steering committee: the research working group and the system implementation group. Both groups will report to the MORAC steering committee.
The research working group will be chaired by the Chief Investigator and will have membership of senior academic staff who will monitor the ongoing design and implementation of the project. This group will meet on a monthly basis.
The System Implementation Group will be chaired by the Head of Cardiology at John Hunter hospital. It will be comprised of clinicians, including the chief investigator, and operational staff to ensure stakeholder communication and address system problems. This group will meet on a fortnightly basis to oversee individual clinical cases and system development. Importantly, this group will also provide feedback to the clinicians reading the ECG and troponin results, at both an individual case level and at the aggregated data level, to maintain optimal adherence to the process.
A clinical events committee (CEC) of experienced cardiologists will also adjudicate all clinical events in a blinded fashion at Monash University. As blinding will not be possible with the system implementation group who review outcomes, scientific rigour demands a blinded review to confirm events. Monash will also provide an ECG core lab reading service. This will provide the most rigorous oversight of the trial. Disagreements on ECG reading will be resolved by consensus at Implementation Group meeting.

<u>Expected impacts of research</u>
This proposal will provide the first comprehensive evidence on the incidence of missed, untreated and under-treated MI, as well as any potential areas for improvements in diagnosis and/or treatment in geographically isolated GP/CMO-led emergency departments. It will thereby inform system improvements in patient care in rural and remote environments. We have previously shown in the HNE LHD that the incidence of MI is increasing in regional and rural settings, and there appears to be increasing rates in aboriginal populations (9). Nationally, the Better Cardiac Care for Aboriginal and Torres Strait Islander Peoples Project

has demonstrated lower rates of angiography and PCI in Indigenous patients with ACS including STEMI (8). Our approach would therefore disproportionately advantage these populations.
The hub and spoke model is an economically efficient and effective way of delivering specialised clinical care and reducing clinical variation across large geographical areas (10). If MORACS is successful at improving health outcomes, and cost-effectiveness is proven, the protocol could rapidly be adapted to other LHDs with rural communities throughout NSW utilising the same centralised service. It is expected that the more hospitals that use a central service, the more cost effective the program will be. The results of our project will enable estimates for how much time is required for the centralised decision support program, and how easily different LHDs can be supported by a central hub. This will inform future scale-up across the state, and whether a single or multiple central hub service(s) would be required to service the entire state.

This research trial will be able to guide key aspects of NSW ACI policy development, clinical care standards and risk stratification for the management of ACS in rural and remotes areas, where lack of specialised cardiology services is a major issue. It will also improve equity of access to evidence-based treatments particularly for patients living in rural locations.

This research aligns with the NSW Health (ACI) reperfusion strategy. Implementation across three rural LHD will inform scalability and effective translation across all rural LHDs in NSW. The ACI reperfusion strategy encourages implementation of new and innovative systems of care across NSW. The ACI cardiology rural working group is the vehicle tasked to inform and implement strategies for rural hospitals to improve care. This TRG aligns with the strategic goals of that group and has its support in application and in translation to clinical practice. Our outcomes will be communicated with the ACI cardiology rural working group via our partners, thence to the ACI cardiac stream. A key strength of our proposal is that we will build on our previous implementation of the STEMI reperfusion program, which is now rolled out state-wide, and will extend this to cover non-STEMI as well. We will optimise patient entry into the existing structures of SCRS and NSW Chest Pain Pathway. Further, the economic evaluations will inform the resources required to implement the intervention, and hence inform a business case to support translation across NSW LHDs.

REFERENCES

1. Chew DP, French J, Briffa TG, Hammett CJ, Ellis CJ, Ranasinghe I, et al. Acute coronary syndrome care across Australia and New Zealand: the SNAPSHOT ACS study. Med J Aust 2013;199(3):185-91.
2. Farshid A, Brieger D, Hyun K, Hammett C, Ellis C, Rankin J, et al. Characteristics and Clinical Course of STEMI Patients who Received no Reperfusion in the Australia and New Zealand SNAPSHOT ACS Registry. Heart, Lung and Circulation. 2016;25(2):132-9.
3. Khan AA, Williams T, Savage L, Stewart P, Faddy S, Ashraf A, et al. Pre-hospital thrombolysis in ST-segment elevation myocardial infarction: a regional Australian experience Medical Journal of Australia. 2016;205(3):121-5.
4. Savage L, Stewart P, Whithead N, Faddy S, Orvad H, Williams T. Missed Acute Myocardial Infarction (MAMI). Heart, Lung and Circulation. 2017;26(Suppl 2):S87.
5. AIHW. Australia's Health 2016. Australian Institute of Health and Welfare. 2016.
6. Economics A. Access Economics. The economic costs of heart attack and chest pain (Acute Coronary Syndrome). 2009.
7. Brieger DB, Redfern J. Contemporary themes in acute coronary syndrome management: from acute illness to secondary prevention. Medical Journal of Australia. 2013;199(3):174-8.
8. AIHW. Better Cardiac Care measures for Aboriginal and Torres Strait Islander people. Second national report 2016. 2016.
9. Davies AJ, Naudin C, Al-Omary M, Khan A, Oldmeadow C, Jones M, et al. Disparities in the incidence of acute myocardial infarction: Long terms trends from the Hunter region. Internal Medicine Journal. 2017;47(5):557-62.
10. Demaerschalk BM, Switzer JA, Xie J, Fan L, Villa KF, Wu EQ. Cost utility of hub-and-spoke telestroke networks from societal perspective. The American journal of managed care. 2013;19(12):976-85.

Attachment 1 - Biographies

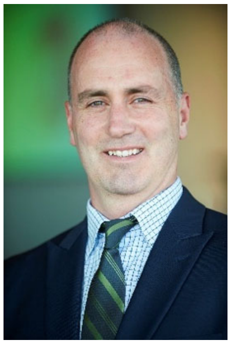

Name, contact details, current appointment, and affiliations

Andrew J. Boyle
Professor of Cardiovascular Medicine, University of Newcastle
Director of Priority Centre for Cardiovascular Health, Hunter Medical Research Institute
Interventional Cardiologist, John Hunter Hospital,
Department of Cardiovascular Medicine
Locked Bag 1, HRMC Newcastle NSW 2310, Australia
Email: andrew.boyle@newcastle.edu.au
Phone: +61 2 4921 4205; Fax: +61 2 4921 4210

Qualifications and background.
MBBS, PhD, FRACP, FACC, FAHA, FSCAI
Prof Boyle is a Cardiologist practising at John Hunter Hospital with extensive experience in cardiovascular research. Trained in Melbourne, Boyle undertook a fellowship at Johns Hopkins in the USA, then was on faculty as a cardiologist and researcher at the University of California San Francisco for over seven years. In Newcastle for four years, he is the founding director of the Priority Clinical Centre for Cardiovascular Health. His research focuses on delivery of cardiac health care in regional and rural settings.

Role on project
Boyle will oversee all of the research aspects of this proposal. He has designed the cluster randomised control trial design, will assist in implementation of the study. Boyle will also oversee data collection from HNE sites and merging of data from the three LHDs. He has communicated with CHeReL and Data Linkage with MBS/PBS/NDI and will co-ordinate this process. He will also be responsible for coordinating statistical analysis, writing the final report and scientific publication of the data in peer-reviewed medical journals.

Experience relevant to the proposal
Boyle has extensive experience across the breadth of clinical trials. He is currently the international PI on a randomised, double-blind, placebo controlled study in 23 sites across Australia and the US. He has led numerous investigator-initiated clinical studies (see below for a selected few) and has been site PI for numerous multi-centre clinical trials. Boyle is on the DSMB of one multi-centre randomised, double-blind clinical trial running across Australia and two smaller clinical trials.
Boyle has studied the outcomes of rural and regional patients in HNE LHD (see below for selected publications), which will be similar to the analyses in the current proposal.

Top 5 publications (relevant to current proposal):

1. Beyer AT, Ng R, Singh A, Zimmet J, Shunk K, Yeghiazarians Y, Ports TA and Boyle AJ. Topical Nitroglycerin and Lidocaine to Dilate the Radial Artery Prior to Transradial Cardiac Catheterization: A Randomized, Placebo-Controlled, Double-Blind Clinical Trial. The PRE-DILATE Study. *International Journal of Cardiology*. 2013;168:2575-2578
2. Pandit J, Gupta V, Boyer N, Yeghiazarians Y, Ports TA and Boyle AJ. Patient and physician perspectives on outcomes weighting in revascularization. The POWR study. *International Journal of Cardiology*. 2014;177:513-514
3. Khan AA, Williams T, Savage L, Stewart P, Faddy S, Ashraf A, Davies AJ, Attia J, Oldmeadow C, Bhagwandeen R, Fletcher P and Boyle AJ. Pre-hospital thrombolysis in ST-segment elevation myocardial infarction: a regional Australian experience *Medical Journal of Australia*. 2016;205:121-125
4. Al-Omary MS, Davies AJ, Khan AA, McGee M, Bastian B, Leitch J, Attia J, Fletcher PJ and Boyle AJ. Heart Failure Hospitalisations in the Hunter New England Area Over 10 years. A Changing Trend. *Heart, Lung and Circulation*. 2017;26:627-630.
5. Davies AJ, Naudin C, Al-Omary M, Khan A, Oldmeadow C, Jones M, Bastian B, Bhagwandeen R, Fletcher P, Leitch J and Boyle A. Disparities in the incidence of acute myocardial infarction: Long terms trends from the Hunter region. *Internal Medicine Journal*. 2017;47:557-562

Name, contact details, current appointment, and affiliations
Peter J Fletcher
Professor of Cardiovascular Medicine, University of Newcastle
Senior Cardiologist, John Hunter Hospital,
Department of Cardiovascular Medicine
Locked Bag 1, HRMC Newcastle NSW 2310, Australia
Email: peter.fletcher@hnehealth.nsw.gov.au
Phone: +61 2 4921 4205; Fax: +61 2 4921 4210

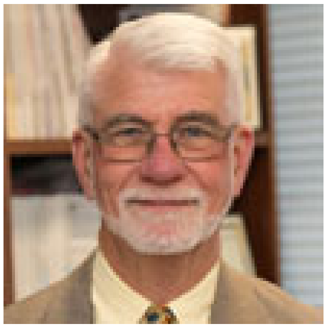

Qualifications and background.
MBBS, PhD, FRACP
Prof Fletcher is a nationally and internationally renowned cardiologist and researcher. He was responsible for pioneering ambulance paramedic-initiated heart attack treatments in Australia.
In 2014, he was recognised in the Queen's birthday honours list, and was awarded Member of the Order of Australia (AM) for his significant service to cardiovascular medicine as a clinician and administrator, heart health programs and medical education. Fletcher is a previous long term chair of the Agency Clinical Innovation (ACI) steering committee. He is the previous Director of Cardiology at John Hunter Hospital. He continues to see patients, supervise junior doctors, and continues to contribute to state-wide cardiology programmes. In recognition of his contribution to cardiovascular medicine, Fletcher was made a Life Member of the National Heart Foundation.

Role on project
Fletcher has extensive experience in system implementation and governance across the state. He will provide senior leadership advice to both the research working group and the system implementation group. Given the role Fletcher has provided on state implementation of cardiology systems, his experience and leadership will be pivotal to the success of this programme.

Experience relevant to the proposal
Fletcher has been a key long term contributor to state systems of care in Cardiology and has designed and implemented key reperfusion systems across the state. He has made significant contributions to the NSW State Cardiac Reperfusion Strategy (SCRS) and has provided long term leadership on the major cardiac programmes both locally and across the state. He designed and directed the development of Pre-Hospital Thrombolysis and lead the introduction of Nurse Administered Thrombolysis – both now adopted across NSW and recipients of Health Ministers Awards and Premier Awards. He has received many local quality awards and state awards for system design, including most recently one for translational research. The implementation of this current project will be modelled on the successful systems that Fletcher designed and implemented.

Top 5 publications (relevant to current proposal):

1. Khan AA, Williams T, Savage L, Stewart P, Faddy S, Ashraf A, Davies AJ, Attia J, Oldmeadow C, Bhagwandeen R, Fletcher P and Boyle AJ. Pre-hospital thrombolysis in ST-segment elevation myocardial infarction: a regional Australian experience *Medical Journal of Australia*. 2016;205:121-125
2. Al-Omary MS, Davies AJ, Khan AA, McGee M, Bastian B, Leitch J, Attia J, Fletcher PJ and Boyle AJ. Heart Failure Hospitalisations in the Hunter New England Area Over 10 years. A Changing Trend. *Heart, Lung and Circulation*. 2017;26:627-630.
3. Davies AJ, Naudin C, Al-Omary M, Khan A, Oldmeadow C, Jones M, Bastian B, Bhagwandeen R, Fletcher P, Leitch J and Boyle A. Disparities in the incidence of acute myocardial infarction: Long terms trends from the Hunter region. *Internal Medicine Journal*. 2017;47:557-562
4. Fletcher P, Stewart P, Savage L. Pros, Cons, and Organization of Prehospital Thrombolysis *Clinical Therapeutics* August 2013; 35: 8: 1058–1063
5. Williams T. Fletcher P. Stewart, P. Faddy, S. Savage, L.(2014) PM211 Pre Hospital Thrombolysis - An Examination of Clinical Outcomes Mar 2014. Heart, Lung and Circulation

Name, contact details, current appointment, and affiliations

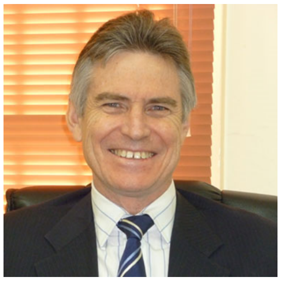

John Kerswell French, Interventional Cardiologist
Prof of Medicine UNSW & WSU (conjoint)
Director, Coronary Care Unit & Cardiovascular Research (Ingham Institute)
Liverpool Hospital, Elizabeth St, Liverpool NSW 2170, AUSTRALIA
office: (02) 87383069 research secretary (02) 87383495; fax: (02) 87383341; mob 0425345103

Qualifications and background.
BMedSc MBChB, MSc PhD, FRACP, FESC, FACC, FCSANZ
Prof French undertook a PhD on oxygen radicals at the University of Adelaide, further cardiology training at Green Lane Hospital New Zealand and a Welcome Trust Postdoctoral Fellowship at University College London, UK. Prior to arriving in Sydney. French was appointed to Green Lane Hospital and the University of Auckland from 1992-2003. French has co-authored >215 peer-reviewed papers. His current research interests include treatments for ST elevation myocardial infarction (STEMI), cardiac biomarkers especially high sensitivity troponins, and diabetes and coronary heart disease. Prof French is CIB on the NHMRC-funded Me-2 trial randomising patients with type-2 MI to coronary angiography or usual care.

Role on project
French has had design input into the MORACS trial. He will be responsible for the oversight of the operation of the trial in the SWSLHD. French will be involved in the statistical analysis, and the development and critical review of the final report and research manuscript(s) to be submitted peer-reviewed medical journals.

Experience relevant to the proposal
French has been on steering committees, and an investigator in numerous randomised controlled trials in STEMI including HERO-1, HERO-2, APEX-AMI, OAT and is currently on the steering committee of STREAM-2 and is national leader. French has served on the clinical endpoints committees (CECs) of several major trials in STEMI including HERO-1, HERO-2, APEX-AMI and PLATO [1/3 patients had STEMI]. Prof French reviews for many journals, and since 2005 has served on the editorial board of the American Heart Journal. French co-authored the 2016 NHF-CSANZ (Cardiac Society of Australia and New Zealand) ACS guidelines (lead writer for the STEMI section).

Top 5 publications (relevant to current proposal):
1. Elliot J, Wang T, Gamble G, Williams M, Matsis P, Troughton R, Hamer A, Devlin G, Mann S, Richards M, French JK, White H, Ellis C. ‘A decade of improvement in the management of New Zealand ST-elevation Myocardial infarction (STEMI) patients: results from the New Zealand Acute Coronary Syndrome (ACS) Audit Group national audits of 2002, 2007 and 2012’. *The New Zealand Medical Journal, 2017; 130(1453):17.*
2. Chew D, Scott I, Cullen L, French JK, Briffa T, Tideman P, Woodruffe S, Kerr A, Branagan M, Aylward P. ‘National Heart Foundation of Australia & New Zealand: Australian Clinical Guidelines for the Management of Acute Coronary Syndromes 2016’. *Heart, Lung and Circ 2016, 25: 895-951.*
3 A.Farshid, D.Brieger[2], K.Hyun, C.Hammett[4] C.Ellis, J.Rankin, J.Lefkovits, D.Chew, J.French 'Characteristics and Clinical Course of STEMI Patients who Received no Reperfusion in the Australia and New Zealand SNAPSHOP ACS Registry. *Heart, Lung and Circ 2016, 25(2):132-139.*
4. Tuan L. Nguyen, John K. French**,** Jarred Hogan, Leia Hee, Daniel Moses, Christian J. Mussap, Rohan Rajaratnam, Craig P. Juergens, Hany R. Dimitri, David A.B. Richards, Liza Thomas. Prognostic value of high sensitivity troponin T after ST-segment elevation myocardial infarction in the era of cardiac magnetic resonance imaging. *Eur Heart J Qual Care Clin Outcomes 2015, 2(3):164-171.*
5. Brieger DB, Chew D, Redfern J, Ellis C, Briffa T, Howell T, Aliprandi-Costa B, Astley C, Gamble G, Carr B, Hammett C, Board N, French JK. Survival after an acute coronary syndrome: 18-month outcomes from the Australian and New Zealand SNAPSHOT ACS Study. *Med J Aust 2015; 203: 368.*

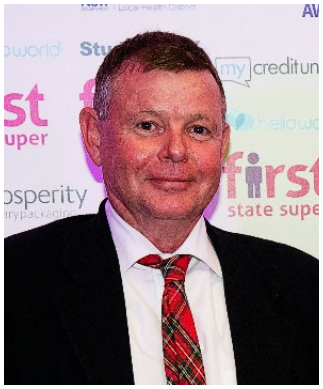

Name, contact details, current appointment, and affiliations

Lindsay Savage
Cardiac Liaison Officer Hunter New England Health
Co-Chair Cardiac Network Agency for Clinical Innovation (ACI)
Registered Nurse John Hunter Hospital
Department of Cardiovascular Medicine
Locked Bag 1, HRMC Newcastle NSW 2310, Australia
Email: Lindsay.Savage@hnehealth.nsw.gov.au
Phone: +61 2 4921 4205; Fax: +61 2 4921 4210

Qualifications and background.

RN. BHSc. MHSc (Research)
Mr Savage is a Nurse Manager working in Cardiology in Hunter New England Health. His research Masters was on comparative methods of delivery of Cardiology Services. He has worked in many capacities within Cardiology and has had a specific interest in the provision of Cardiology care in a rural setting. Savage has played an active role in the design and delivery of services both within Hunter New England Health and NSW through an active role within the ACI.

Role on project

Savage will oversee all of the operational aspects of this proposal. He has helped design the reading service and has been active in the development of automated methods of ECG and troponin acquisition. Lindsay will also directly manage the reading service on Monday to Friday.
Savage will be responsible for the communication and roll-out of the current proposal across all the LHD. He will work closely with the project manager to ensure that the research proposal flows smoothly from the operational implementation.

Experience relevant to the proposal

Savage has extensive experience in the design and development of clinical systems in Cardiology. He was the project lead for HNELHD in the development of Pre-Hospital Thrombolysis and one of the project leads for Nurse Administered Thrombolysis – both now adopted across NSW and recipients of Health Ministers Awards and Premier Awards. He was the project lead for the integrated Cardiology reports database which provides linked Cardiology reports including ECG across all sites in Hunter New England. He has received many local quality awards for system design including most recently one for translational research.
The implementation of this current project will be modelled on the successful systems outlined above.

Top 5 publications (relevant to current proposal):

1. Fletcher P, Stewart P, Savage L. Pros, Cons, and Organization of Prehospital Thrombolysis ***Clinical Therapeutics*** August 2013; 35: 8: 1058–1063
2. Khan AA, Ashraf A, Baker, D, Savage L, Collins N. Clozapine and Incidence of Myocarditis and Sudden Death & Long Term Australian Experience 238 · March **2017 *International journal of cardiology***
3. Khan AA, Williams T, Savage L, Stewart P, Faddy S, Ashraf A, Davies AJ, Attia J, Oldmeadow C, Bhagwandeen R, Fletcher P and Boyle AJ. Pre-hospital thrombolysis in ST-segment elevation myocardial infarction: a regional Australian experience ***Medical Journal of Australia***. 2016; 205:121-125
4. Savage L. Whitehead, N. Stewart, P. Williams, T Missed Acute Myocardial Infarction (MAMI) ***Heart, Lung and Circulation*** 26:S87 · December 2017
5. Khan A, Williams T, Savage L. Boyle, A. Pre-hospital thrombolysis in ST-segment elevation myocardial infarction: a regional Australian experience: real world long term follow up Apr 2016 Journal of the American College of Cardiology

Name, contact details, current appointment, and affiliations

John Attia
Professor of Medicine and Clinical Epidemiology at the University of Newcastle
Director of the Clinical Research Design, IT, and Statistical Support (CReDITSS) Unit
Director of general medicine at John Hunter Hospital
CReDITSS Unit: Level 3, Hunter Medical Research Institute University of Newcastle Callaghan 2308 NSW Australia
Email: john.Attia@newcastle.edu.au
Phone: (02) 40420515. Fax (02) 40420001

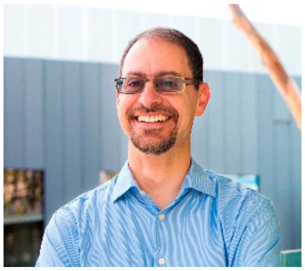

Qualifications and background

MD, PhD, FRACP, FRCPC BSc, MSc

Prof Attia is Professor of Medicine and Clinical Epidemiology at the University of Newcastle and has expertise in population, clinical, molecular and genetic epidemiology. Professor Attia trained at McMaster University (Canada) in clinical medicine with a specialty in general internal medicine (MD) and obtained his fellowship with the Royal College of Physicians of Canada and the Royal Australasian College of Physicians. He also obtained a BSc in Physiology (Faculty scholar at McGill University), a MSc in Epidemiology (McMaster University), and a 5 year MRC scholarship to complete his PhD in Molecular Genetics (University of Toronto). He has been listed on the NHMRC register of Evidence-Based Medicine experts and has provided epidemiological expertise to the Therapeutic Goods Administration and currently to the Medical Services Advisory Committee. He is currently academic director of general medicine at John Hunter Hospital responsible for the advanced training program, as well as director of the Clinical Research Design, IT, and Statistical Support (CReDITSS) Unit, a unit that provides epidemiological and statistical methodological advice to clinical researchers.

Role on project

Attia has assisted extensively in the planning phase of this project including trial design, sample size calculation, statistical methods and analysis. Attia's vast clinical, epidemiology and research background will ensure he is a key member research team. CReDITSS will co-ordinate statistical analysis and provide ongoing support to this study. Attia will also be a key member of the research steering committee who will oversee and monitor the ongoing design and implementation of the project.

Experience relevant to the proposal

Attia's research experience is significant, including 505 over career, 265 over last 5 years (2013-2017) Averaging >1 publication/week since 2011,15,760 citations over career, 11,999 over last 5 years, with an h-index of 67 (Google Scholar, accessed Dec 22, 2017),96 conference abstracts, 7 government reports and 6 book chapters. Winner of the Research Excellence Award from the Faculty of Health, University of Newcastle (2012). Winner of the Dudley Homer Vose Award from the National Heart Foundation for best grant (2005). Attia is reviewer for leading peer review journals including, Am J Epidemiol, Int J Epidemiol, J Clin Epidemiol, JAMA, Stroke, PLoS One, PLoS Genetics. He has over $24.3 million in competitive, peer-reviewed grant income over career.

Top 5 publications (relevant to current proposal):

1. Ren S, Newby D, Li SC, Walkom E, Miller P, Hure A, Attia J. Effect of the adult pneumococcal polysaccharide vaccine on cardiovascular disease: a systematic review and meta-analysis. *Open Heart.* 2015; 2(1):1-9
2. Briggs S, Pearce R, Dilworth S, Higgins I, Hullick C, Attia J. Clinical pharmacist review: A randomised controlled trial. *EMA - Emergency Medicine Australasia*. 2015; 27(5):419-26
3. Robertson J, Pearson SA, Attia JR. How well do NSW hospital data identify cases of heart failure? *Medical Journal of Australia*. 2014; 200(1):25
4. Lu Y, Hajifathalian K, Ezzati M, Woodward M, Rimm EB, Danaei G. Metabolic mediators of the effects of body-mass index, overweight, and obesity on coronary heart disease and stroke: a pooled analysis of 97 prospective cohorts with 1.8 million participants. *Lancet*. 2014 Mar 15; 383(9921):970-83.
5. Al-Omary MS, Davies AJ, Khan AA, McGee M, Bastian B, Leitch J, Attia J, Fletcher PJ, Boyle AJ. Heart Failure Hospitalisations in the Hunter New England Area over 10 years. A Changing Trend. *Heart Lung Circ*. 2017; 26 (6): 627-630

Name, contact details, current appointment, and affiliations

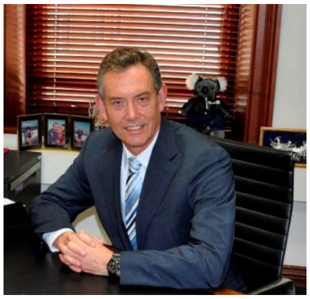

James Leitch
Director of Cardiovascular Medicine: John Hunter Hospital.
Clinical Lead Cardiology Stream HNELHD
Associate Professor Cardiology. University of Newcastle
Department of Cardiovascular Medicine
Locked Bag 1, HRMC Newcastle NSW 2310, Australia
Email: James.Leitch@hnehealth.nsw.gov.au
Phone: +61 2 4921 4205; Fax: +61 2 4921 4210

Qualifications and background

MB, BS, FRACP

Dr Leitch graduated Sydney University 1980 MB BS and undertook intern, resident and internal medicine training at Royal Newcastle Hospital. Advanced training in cardiology at Royal Prince Alfred Hospital, Sydney followed by 1 year research fellowship in cardiology at RPA. Training in electrophysiology and pacing London Ontario Canada 1989-91 under the mentorship of Dr George Klein.

Appointed Staff Specialist Cardiology at John Hunter Hospital 1991, he is currently Director of Cardiology John Hunter Hospital. Leitch established an electrophysiology service at John Hunter Hospital which provides specialized services to patients of the Hunter, North Coast and Gosford. The service has a strong focus of providing high level services to patients in remote and regional areas and conducts outreach services in Taree, Forster, Singleton, Port Macquarie and Gosford.

Role on project

Leitch will provide clinical supervision and support. He will provide organisational oversight of the trial implementation as the chair of System Implementation Group. Leitch will also provide governance support to the project. Through his position as chair of the cardiac stream he will provided logistical support to all hospitals and provide a key link to all medical staff councils through his dual roles within Cardiology. He will also co-ordinate communication to the Executive Leadership Team (ELT).

Experience relevant to the proposal

Leitch has extensive experience across research and clinical systems. He is a nationally recognised Cardiologist. He has previously authored guidelines for the prevention, detection and management of chronic heart failure in Australia (2006). He has published 87 peer reviewed articles and book chapters including first author publications in Circulation and JACC and published more than 110 abstracts including an abstract selected for Ralph Reader prize competition CSANZ and winning the poster prize 1996. He has total citations of 3836, with an H index 30 and an I10 index 46. He has been successful in attracting grant support from NHMRC and the Heart foundation and participated in numerous commercially funded research projects. Leitch is a nationally recognised cardiologist, particularly in the field of electrophysiology and pacing.

Top 5 publications (relevant to current proposal):

1. Al-Omary MS, Davies AJ, Khan AA, McGee M, Bastian B, Leitch J, Attia J, Fletcher PJ, Boyle AJ. Heart Failure Hospitalisations in the Hunter New England Area over 10 years. A Changing Trend. *Heart Lung Circ*. 2017; 26 (6): 627-630
2. Davies AJ, Naudin C, Al-Omary M, Khan A, Oldmeadow C, Jones M, Bastian B, Bhagwandeen R, Fletcher P, Leitch J and Boyle A. Disparities in the incidence of acute myocardial infarction: Long terms trends from the Hunter region. *Internal Medicine Journal*. 2017;47:557-562
3. Wilsmore B, Leitch J. Remote monitoring of medical devices in Australia. *Medical Journal of Australia.*2017; 206 (2): 62-63
4. Davies AJ, Gunaruwan P, Collins N, Barlow. M, Jackson N, Leitch J. Persistent iatrogenic atrial septal defects after pulmonary vein isolation: long-term follow-up with contrast transesophageal echocardiography. *Journal of Interventional Cardiac Electrophysiology* (2016) 48(1*)*: 99-103
5. Davies AJ, Jackson NJ, Barlow M, Leitch J. Long Term Follow-up of Pulmonary Vein Isolation Using Cryoballoon Ablation *Heart, Lung and Circulation* 25(3) 290-295

Name, contact details, current appointment, and affiliations

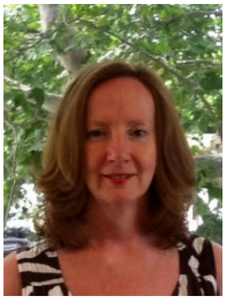

Kerry Inder
Associate Professor of Nursing and Deputy Head of School for Research,
School of Nursing University of Newcastle
The University of Newcastle (UoN) Richardson Wing, room RW1-38 University Drive, Callaghan NSW 2308
Email: Kerry.Inder@hnehealth.nsw.gov.au
Phone: +61 2 40420522; Fax: +61 2 4921 4210

Qualifications and background.

PhD, GRAD DIP CLIN EPID, BN, COR CARE, RN

Assoc Prof Inder's research experience and interests are in chronic disease, mental health and wellbeing, suicidal behaviour, ageing well and rural health. Inder has published three reports and 60 peer reviewed journal articles; with 843 citations (681 since 2013). H-Index = 17; i10-index is 32. Kerry is currently supervising 15 higher degree research students (11 PhD Candidates and 4 Masters Candidates); across Nursing, Gender and Health, Community Medicine and Clinical Epidemiology and Psychiatry programs; 8 as principal supervisor. Inder has supervised six students to completion. Kerry has received approximately $1.2 million in research funding for 17 projects. Inder has been chief investigator on two nationally competitive research grants with the Australian Coal Association Research Program valued at over $640,000 and associate investigator on the NHMRC Centre for Research Excellence in Severe Asthma (2015-19) valued at $2.3m. Inder has been honoured as the recipient of the School Research Supervision Excellence award (2017), Faculty of Health Research Staff Excellence Award (2012) and HMRI Xstrata Coal research fellowship (2010-2013).

Role on project

Inder will assist with implementation of the design, training of staff involved in data collection and interpretation and dissemination of results.

Experience relevant to the proposal

Inder was previously employed as a Clinical Nurse Consultant (Level 3) for Cardiac Rehabilitation at John Hunter Hospital, Newcastle. Inder has a 20-year background in clinical nursing with extensive experience in coronary care and cardiac rehabilitation. Inder's PhD evaluated the health outcomes of a nurse-led outpatient cardiac rehabilitation program in terms of survival and unplanned re-hospitalisation. She therefore brings insight relevant to system implementation and post discharge events in cardiac patients.

Top 5 publications (relevant to current proposal):

1. Gunathilake R, Oldmeadow C., McEvoy M, Kelly B, Inder K., Schofield P, Attia J. Mild hyponatremia is associated with impaired cognition and falls in community-dwelling older persons. *J Am Geriatr Soc*, 2013; 61:10, 1838-1839
2. Carey, M., Jones, K., Meadows, G., Sanson-Fisher, R., D'Este, C., Inder, K., Yoong, S., Russell, G.. Accuracy of general practitioner unassisted detection of depression. *Aust N Z J Psychiatry* 2014; 48:6, 571-578
3. Gunathilake R, Oldmeadow C, McEvoy M, Kelly B, Inder KJ, Schofield P, Nair K, Attia The association between obesity and cognitive function in older persons: how much is mediated by inflammation, fasting plasma glucose and hypertriglyceridemia? Journals of Gerontology: Biological Sciences and Medical Sciences. 2016; 71:912:1603-1608
4. Carey M, Boyes A, Noble N, Waller A, Inder KJ. Validation of the PHQ-2 against the PHQ-9 for detecting depression in a large sample of Australian general practice patients. Australian Journal of Primary Health Research 2015
5. Handley, T. E., Kay-Lambkin, F. J., Inder, K. J., Attia, J. R., Lewin, T. J., & Kelly, B. J. (2014). Feasibility of internet-delivered mental health treatments for rural populations. Soc Psychiatry Psychiatr Epidemiol 2014;49:2, 275-282.

Name, contact details, current appointment, and affiliations

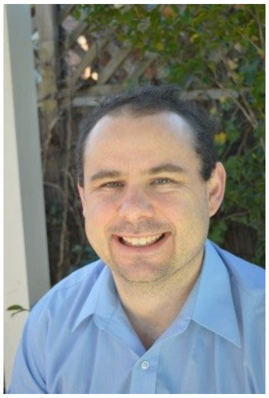

Trent Williams
Clinical Nurse Specialist John Hunter Hospital/ Clinical Risk Officer
Department of Cardiovascular Medicine
Locked Bag 1, HRMC Newcastle NSW 2310, Australia
Email: trent.williams@hnehealth.nsw.gov.au
Phone: +61 2 4921 4205; Fax: +61 2 4921 4210

Qualifications and background.
Current PhD Candidate School of Nursing and Midwifery University of Newcastle, Australia
Bachelor of Nursing, Graduate Certificate Acute Cardiology, Certificate IV Occupational Health and Safety

Mr Williams is a Clinical Nurse specialist employed at John Hunter Hospital with extensive long term clinical experience in cardiology. He is a graduate of the University of Newcastle where he is currently undertaking his PhD examining adverse events and complications in a cardiology setting. The clinical risk officer position is responsible for the collection of area adverse events and data collection. In addition the management of the John Hunter Hospital STEMI database which he conceived and maintains. Williams also has conceived and implemented the rural ACS database which provides the area prospective results of ECG and pathology of ACS patients who present across the district. He has audited compliance of all HNE hospitals and is currently collaborating on national research trials within Cardiology and across specialities. Williams has been an invited speaker to national conferences on STEMI systems of care. Williams has a keen interest in clinical cardiology and system of care monitoring. He was recently awarded the BRICS's research award for nursing research.

Role on project
Williams will assist with coordination of the project, educational aspects of training including data collection due to his long standing knowledge of data systems within the district. He has assisted with the development of the system of care for this project and will help formulate data collection tools for the study. He will play a significant role in implementation of the study. He will work closely with both the research and operational teams to ensure the effective rollout and monitoring of the service.

Experience relevant to the proposal
Williams has extensive experience in the implementation and monitoring of clinical systems within Cardiology. He was involved in the implementation of the prehospital thrombolysis programme and also the implementation and monitoring of the Pre- Hospital Activation of Primary Angioplasty service within the district. He recently led a nurse driven research trial of complications funded from a grant. Williams has made a significant contribution to the implementation of rural ACS strategies. Williams has been the recipient of local quality awards and was a member of the team who was awarded the state Translational research award.

Top 5 publications (relevant to current proposal):
1. Khan AA, Williams T, Savage L, Stewart P, Faddy S, Ashraf A, Davies AJ, Attia J, Oldmeadow C, Bhagwandeen R, Fletcher P and Boyle AJ. Pre-hospital thrombolysis in ST-segment elevation myocardial infarction: a regional Australian experience *Medical Journal of Australia*. 2016;205:121-125
2. Savage L. Whitehead N. Stewart P. Williams, T.2017 Missed Acute Myocardial Infarction (MAMI) Heart, Lung and Circulation 26:S87 ·
3. Khan AA, Williams T, Savage L. Boyle A. Pre-hospital thrombolysis in ST-segment elevation myocardial infarction: a regional Australian experience real world long term follow up. Apr 2016 Journal of the American College of Cardiology.
4. Williams T. Fletcher P. Stewart, P. Faddy, S. Savage, L.2014; PM211 Pre Hospital Thrombolysis - An Examination of Clinical Outcomes Mar 2014. Heart, Lung and Circulation
5. Williams T. Savage L. Inder K. Collins N. 2014 Impact of Change to Vascular Access Route on Patient Outcomes for PCI Following Thrombolysis for AMI. Global Heart,9 (1), S e334.

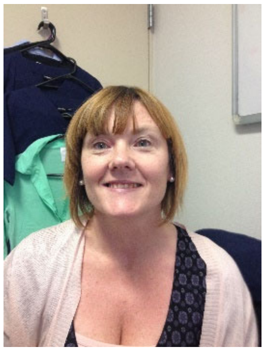

Name, contact details, current appointment, and affiliations

Dawn McIvor
Clinical Nurse Consultant: John Hunter Hospital.
Co-ordinator Cardiology Stream HNELHD
Department of Cardiovascular Medicine
Locked Bag 1, HRMC Newcastle NSW 2310, Australia
Email: Dawn.Mcivor@hnehealth.nsw.gov.au
Phone: +61 2 4921 4205; Fax: +61 2 4921 4210

Qualifications and background.

Masters Philosophy, BSC Hons Health Studies, Post Graduate Diploma mentorship, Registered General Nurse.

McIvor is the past President of the Cardiac Rehabilitation Association NSWACT. She is currently secretary of this organisation and has been a previous member of the executive committee of ACRA. McIvor played an integral role on the NSW cardiac rehabilitation working group developing MDIs for cardiac rehabilitation. McIvor has been on the writing groups for a number of state-wide guidelines including cardiac monitoring and deactivation Implantable cardiac defibrillators (ICD).

Role on project

Ms McIvor is a senior Clinical Nurse Consultant and has extensive experience in the support of large scale cardiology services locally and across the state. She has assisted with the implementation of both acute and chronic systems of care, and the provision of education and support of all clinical staff. McIvor has an extensive background of monitoring and reviewing of systems both locally and internationally. McIvor will play a key role in disseminating results and providing ongoing communication throughout the district through her role as the co-ordinator of the cardiology stream.

Experience relevant to the proposal

Twenty years of both acute and chronic cardiology experience in a variety of settings. McIvor played a key role in the rollout of Nurse Administered Thrombolysis – which has been adopted across NSW and was the recipient of Health Ministers Awards and Premier Awards. McIvor has experience in implementing new systems of care in the rural environment. McIvor has been a long term facilitator of education and supporting clinical staff around the provision of acute and chronic cardiology care. Through her role within HNELHD she was involved in both the conceiving and implementation of clinical guidelines, education programs related to cardiology for all clinical staff. She played an active role in developing and implementing new model of care for cardiac rehabilitation, Heart failure and interventional procedural services.

Top 5 publications (relevant to current proposal):

1. Outcomes following heart failure hospitalization in regional Australian setting between 2005 and 2014: Al-Omary M, Khan A, Davies, A Fletcher P, McIvor D et al. ESC Heart failure (2017)
2. Screening for obstructive sleep apneoa in cardiac rehabilitation: A position statement from the Australian Centre for Heart Health and the Australian Cardiovascular Health and Rehabilitation Association: Le Grande M, Neubeck L, Murphy B, McIvor D, Lynch D, European Journal of Preventative Cardiology vol. 23, 14: pp. 1466-1475 (2017)
3. Savage L, Williams T, Fletcher P, Stewart P, McIvor D, Orvad, H. Systems of Reperfusion for STEMI in Hunter New England NSW. 2014; Global Heart 9:1 e281

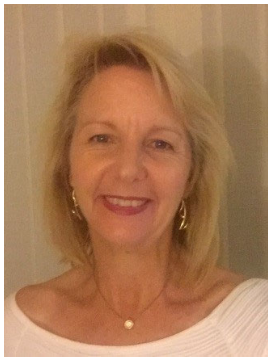

Name, contact details, current appointment, and affiliations

Helen Orvad
Clinical Nurse Consultant Northern Hunter New England Local Health District
Department of Cardiovascular Medicine
Dean St, North Tamworth NSW 2340 Tamworth Hospital
Email: Helen.Orvad@hnehealth.nsw.gov.au
Phone: +61 2 4921 4205; Fax: +61 2 4921 4210

Qualifications and background.

Bachelor of Nursing, Masters of Nursing: Cardiac

Ms Orvad is a senior Clinical Nurse consultant and has as extensive experience at local, state and national cardiology bodies. Orvad has been New South Wales College of Nursing, the Cardiac Society of Australia and New Zealand, the Australian and New Zealand Interventional Nurses Council and National Rural Health Alliance. Orvad has significant organisational and administration experience including, Tamworth Rural Referral Hospital Cardiology Clinical Executive, HNELHD Cardiac Stream Acute Working Group, HNELHD Cardiac Stream Heart Failure reference group, Agency for Clinical Innovation Cardiac Network, Co-chair Agency for Clinical Innovation Rural Cardiac Network, Heart Foundation Rural Working Group, Lighthouse Project Steering Committee, and the Rheumatic Heart Disease Working Party HNELHD

Role on project

A significant number of hospitals in the research will be located in the North of HNELHD. Orvad's clinical implementation experience will be vital. She will oversee all of the operational aspects of this proposal in the north which is a large geographical area of this project She has helped design the reading service and has been active in the development of automated methods of ECG and troponin acquisition. Orvad will work closely with the project manager to ensure that the research proposal flows smoothly from the operational implementation in the northern LHD. Orvad will also assist with the dissemination of the results to members of the northern rural health team.

Experience relevant to the proposal

The role of rural Clinical Nurse Consultant for Cardiology involves the clinical and observational management of STEMIs within 18 rural sites within HNELHD. Orvad has extensive rural Cardiology experience. Orvad has a significant background in the monitoring of clinical variation and operational issues from a whole of district perspective. Orvad has played a significant role in the governance and co-ordination of adverse events within HNE-LHD. She has played a key role in the roll out of other HNE projects in a collaborative model, engaged stakeholders, evaluated project outcomes and engaged with Clinical Governance where required. Orvad's knowledge of rural cardiology both locally and across the state will assist with the implementation of this project across a number of LHD'S.

Top 5 publications (relevant to current proposal):

1. Savage L, Williams T, Fletcher P, Stewart P, McIvor D, Orvad, H. Systems of Reperfusion for STEMI in Hunter New England NSW. 2014; Global Heart 9:1 e281

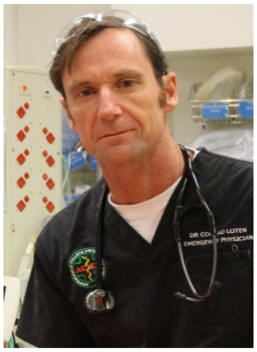

Name, contact details, current appointment, and affiliations

Conrad Loten
Staff Specialist in Emergency Medicine.
Deputy Director, Division of Emergency Medicine
Emergency Department John Hunter Hospital
Locked Bag 1, HRMC Newcastle NSW 2310, Australia
Email: Conrad.loten@hnehealth.nsw.gov.au
Phone: +61 0427278867 Fax: +61 2 4921 4210

Qualifications and background

MBChB Otago University, 1989, Dip RACOG Fellow Australasian College Emergency Medicine, 1996. Loten is a practicing emergency staff specialist employed at John Hunter Emergency Department. Loten has a keen interest in the delivery of cardiology care in the Emergency Department and has previously done extensive work in the management of Acute Coronary Syndrome across HNE-LHD and published in peer reviewed journals. Loten has previously won research awards including 2007 AMA /Wyeth Prize for best paper in MJA 2007, and 2006 Best trainee paper at ACEM.

Role on project

Loten is a highly respected senior emergency physician who will provide clinical input into the design and implementation of this programme. He is the “clinician champion” of the ED diagnosis and treatment of ACS. Loten’s previous work in Troponin collection and clinical experience will be integral components of this programme. Loten has a strong and long-term commitment to medical education within the district (see below) and will lead the educational initiative with the GPs that staff rural and remote EDs at the commencement of the project. This will be pivotal to the education and support of rural and regional medical staff during the implementation of this programme. Loten’s reputation across the district will assist with interpretation and dissemination of results to Emergency Departments across all districts.

Experience relevant to the proposal

Loten has extensive experience in all research methods, he is currently a site supervisor for a number of multi centre Randomised Control Trials and large observational studies. He will assist with monitoring of this project from both a research and emergency clinical care view point. Loten has studied the outcomes of a number of important aspects of this study, in particular point of care Troponin (see below) in the study population. He has considerable experience in a variety of research methods, as well providing clinical support, education and system implementation advice from an Emergency physician perspective. Loten has lead the districts Emergency Medicine Education & Training (EMET) Outreach program, which is a federally funded project that seeks to increase clinical skills of rural practitioners and teaches the importance of team work and efficient communication. These 2 programmes will complement each other to increase the skills of rural health professionals in acute cardiology.

Top 5 publications (relevant to current proposal):

1. A randomised controlled trial of hot water (45 degrees C) immersion versus ice packs for pain relief in bluebottle stings. Loten C, Isbister, G et al. *Medical Journal of Australia*. 2006 Apr 3;184(7):329-33
2. Point of care troponin decreases time in the emergency department for patients with possible acute coronary syndrome: a randomised controlled trial. Loten C, Attia J, Hullick C, Marley J, McElduff P. *Emerg Med J.(BMJ)* 2010 Mar;27(3):194-8
3. Validation of a point of care troponin assay in real life Emergency Department conditions. Loten C, Attia J, Hullick C, Marley J. *Emerg Med Australas*. 2009 Aug; 21(4):286-92
4. Adverse outcomes following emergency department discharge of patients with possible acute coronary syndrome. Loten C, Isbister G, Jamcotchian M, Hullick C, MacElduff P, Attia J, Marley J. *Emerg Med Australas*. 2009 Dec; 21(6):455-64.
5. High-flow oxygen in patients undergoing procedural sedation in the emergency department: A retrospective chart review. Thomson D, Cowan, T, Loten C, Botifeld C, Holliday L, Attia, J. Emergency medicine Australasia; 2016 29(1) 33-39
