# Supplementary material for: Management of Acute Coronary Syndromes in Patients in Rural Australia: The MORACS Randomized Clinical Trial
Source: JAMA Cardiol. 2022 May 25;7(7):690–8. doi: 10.1001/jamacardio.2022.1188 (PMC10881213; doi:10.1001/jamacardio.2022.1188)
Supplement: Supplement 2. — eTable 1. Hospitals included in the MORACS Trial eTable 2. All patients baseline characteristics eTable 3. STEMI patient demographics eTable 4. Triage codes used for recruitment in MORACS eTable 5. Primary and secondary outcomes eTable 6. Additional outcomes – reperfusion [file jamacardiol-e221188-s002.pdf]

Supplemental Online Content

Dee F, Savage L, Leitch JW, et al. Management of acute coronary syndromes in patients in rural Australia: the MORACS randomized clinical trial. *JAMA Cardiol*. Published online May 25, 2022. doi:10.1001/jamacardio.2022.1188

eTable 1. Hospitals included in the MORACS Trial

eTable 2. All patients baseline characteristics

eTable 3. STEMI patient demographics

eTable 4. Triage codes used for recruitment in MORACS

eTable 5. Primary and secondary outcomes

eTable 6. Additional outcomes – reperfusion

This supplemental material has been provided by the authors to give readers additional information about their work.

© 2022 Dee F et al. *JAMA Cardiology.*

eTable 1. Hospitals included in the MORACS Trial

Armidale
Barraba MPS
Bingara MPS
Boggabri
Cessnock
Denman MPS
Dungog
Glenn Inness
Gloucester Hospital
Gunnedah
Guyra MPS
Inverell
Kurri Kurri
Manilla
Merriwa
Moree
Muswellbrook
Narrabri
Quirindi
Scott Memorial Hospital (Scone)
Singleton
Tenterfield
Tingha
Tomaree Hospital
Vegetable creek
Walcha
Warialda
Wee Waa
Wilson Memorial Hospital (Murrurundi)

© 2022 Dee F et al. *JAMA Cardiology.*

eTable 2. All patients baseline characteristics

All patients		Control (n=3342)	Intervention (n=2907)
Gender	Female	1639 (49%)	1361 (47%)
Aboriginality	Aboriginal/TSI	521 (16%)	414 (14%)
ASGS Regionality (2016)	Major cities	409 (12%)	123 (4.2%)
	Inner regional	1451 (43%)	1862 (64%)
	Outer regional	1457 (44%)	908 (31%)
	Remote	16 (0.5%)	3 (0.1%)
	Very remote	3 (0.1%)	1 (0.0%)
IRSAD quartile (2016)	Quartile 1	1012 (30%)	117 (4.0%)
	Quartile 2	2211 (66%)	2725 (94%)
	Quartile 3	92 (2.8%)	40 (1.4%)
	Quartile 4	21 (0.6%)	15 (0.5%)
Prior myocardial infarction (12 months)	Yes	467 (14%)	374 (13%)
Prior stent	Yes	317 (9.5%)	277 (9.5%)
Prior CABG	Yes	141 (4.2%)	122 (4.2%)
Diabetes	Yes	511 (15%)	373 (13%)
Age at presentation	mean (SD)	60.8 (16.3)	58.9 (16.1)

© 2022 Dee F et al. *JAMA Cardiology.*

eTable 3. STEMI patient demographics

STEMI Patients		Usual Care (n=77)	Intervention (n=46)
Age at admission	mean (SD)	65.1 (12.8)	61.2 (10.7)
Gender	Male n(%)	60(78)	36(78)
Aboriginality n(%)	Aboriginal not Torres Strait Islander	13 (17)	3 (7)
	Torres Strait Islander not Aboriginal	0	1(2)
	Both	1(1)	0
	Neither	62(82)	41(91)
Prior myocardial infarction (12 months)	n(%)	2 (23)	2 (4)
Prior stent	n(%)	6 (8)	5 (11)
Prior CABG	n(%)	2 (3)	2 (4)
Prior diabetes	n(%)	18 (23)	9 (20)

STEMI Patients		Usual Care (n=77)	Intervention (n=46)
ED presentation in prior 21 days	n(%)	7(9)	1(2)
Mode of arrival n(%)	Ambulance	20(26)	10(22)
	Private car	57(73)	36(78)
	Other	1(1)	0
Triage Category n(%)	ATS1 Resuscitation	2(3)	3(7)
	ATS2 Emergency	69(88)	40(87)
	ATS3 Urgent	4(5)	3(7)
	ATS4 Semi-urgent	3(4)	0
	ATS5 Non-urgent	0	0

© 2022 Dee F et al. *JAMA Cardiology.*

eTable 4. Triage codes used for recruitment in MORACS

CODE	DESCRIPTION
I20.0	Unstable Angina (UAP)
I20.9	Angina Pectoris
I20.9	Chest Pain, Ischaemic (Angina)
I20.9	Ischaemic Chest Pain (Angina)
I46.9	Cardiac Arrest
R07.4	Chest Pain (non-traumatic)
I20.0	Unstable Angina (UAP) *
I20.9	Pain in Chest, Ischaemic (Angina)*
I20.9	Angina Pectoris (AP)*
I46.9	Cardiac Arrest
R07.4	Pain - Chest (Cardiac Origin)
I20.0	Acute Coronary Syndrome (ACS)
R07.4	Chest Pain - Atypical
R10.1	Pain - Epigastric
R10.4	Pain - Abdominal
R06.0	Respiratory - SOB

Denotes ICD codes merged and no longer individually available in the ICD10 triage code selection

© 2022 Dee F et al. *JAMA Cardiology.*

eTable 5. Primary and secondary outcomes

Variable		Usual care (n=78)	MORACS Intervention (n=46)	P value
**Primary outcome**				
Failure to recognise STEMI	n(%)	27(35)	0	0.001
**Secondary outcomes**				
All-cause mortality	n(%)	6(8)	1(2)	0.198
Hospital length of stay (days)	median (Q1, Q3)	4 (3, 6)	3 (2, 4)	0.089
30 day readmission	n(%)	7(9)	8(17)	0.253
Reperfusion strategy				0.002
	Thrombolysis and transfer n(%)	40(51)	35(76)	
	Reperfusion ineligible n(%)	3(4)	8(17)	
	Transfer for Primary PCI n(%)	12(15)	1(2)	
	Spontaneous reperfusion n(%)	1(1)	2(4)	
	No reperfusion therapy n(%)	22(28)	0	
Time to reperfusion (minutes)	Thrombolysis	50 (39, 100)	58 (35.4, 75.5)	0.251
	Primary PCI	140 (123, 195)	268	n/a

© 2022 Dee F et al. *JAMA Cardiology.*

eTable 6. Additional outcomes – reperfusion

Variable		Missed diagnosis	Correct diagnosis	P Value
Reperfusion time by diagnosis (mins)	median (Q1, Q3)	140 (94, 396)	59 (38, 104)	0.057
Reperfusion offered (includes all Rx <12hrs)	n(%)	8(30)	83(98)	0.000

© 2022 Dee F et al. *JAMA Cardiology.*
