# Supplementary material for: Management of Acute Coronary Syndromes in Patients in Rural Australia: The MORACS Randomized Clinical Trial
Source: JAMA Cardiol. 2022 May 25;7(7):690–8. doi: 10.1001/jamacardio.2022.1188 (PMC10881213; doi:10.1001/jamacardio.2022.1188)
Supplement: Supplement 3. — Data sharing statement [file jamacardiol-e221188-s003.pdf]

Data Sharing Statement

Dee. Management of Acute Coronary Syndromes in Patients in Rural Australia. *JAMA Cardiol*. Published May 25, 2022. doi:10.1001/jamacardio.2022.1188

Data
Data available: No

Additional Information
Explanation for why data not available: Reasonable requests for data sharing will be considered by contacting the corresponding author.
